# EHMTI-0063. The migraine disability assessment (MIDAS) questionnaire: translation, validation and reliability of Bahasa Melayu version

**DOI:** 10.1186/1129-2377-15-S1-D56

**Published:** 2014-09-18

**Authors:** MM Shaik, NB Hassan, HL Tan, SH Gan

**Affiliations:** 1 Human Genome Centre, Universiti Sains Malaysia, Kubang Kerian, Malaysia; 2 Department of Pharmacology, Universiti Sains Malaysia, Kubang Kerian, Malaysia

## Introduction

MIDAS has become a popular and useful tool for evaluating migraine-related disability worldwide.

## Aim

The study was designed to determine the validity and reliability of the Bahasa Melayu version (MIDAS-M) of the Migraine Disability Assessment (MIDAS) Questionnaire.

## Methods

The patients attending the Neurology Clinic, Hospital Universiti Sains Malaysia, Kubang Kerian, Kelantan, Malaysia, were screened against the inclusion and exclusion criteria. Patients were eligible for the study if they had been diagnosed with migraine for more than six months. Standard forward and back translation procedures were used to translate and adapt the MIDAS Questionnaire to produce the Bahasa Melayu version. The translated Malay version was tested for face and content validity. Validity and reliability testing were further conducted with 100 migraine patients (1st administration) followed by a retesting session 21 days later (2nd administration).

## Results

A total of 100 patients between 15-60 years of age were recruited. The majority of the patients were single (66%), students (46%) and had a severe disability (46% MIDAS grade IV). The sample was adequate according to the Kaiser-Meyer-Olkin value of 0.75. The Cronbach's alpha values were 0.84 (1st administration) and 0.80 (2nd administration). The test-retest reliability for the total MIDAS score was 0.73, indicating that the MIDAS-M questionnaire is stable; for the five disability questions, the test-retest values ranged from 0.77 to 0.87.

## Conclusion

The MIDAS-M questionnaire is comparable with the original English version in terms of validity and reliability and may be used for the assessment of migraine in clinical settings.

No conflict of interest.

